# Free Amino Acids and Biogenic Amines Profiling and Variation in Wild and Sub-Endemic Cardueae Species from Sardinia and Corse

**DOI:** 10.3390/plants12020319

**Published:** 2023-01-10

**Authors:** Arianna Marengo, Larissa Silva Maciel, Cecilia Cagliero, Patrizia Rubiolo, Koit Herodes

**Affiliations:** 1 Dipartimento di Scienza e Tecnologia del Farmaco, Università di Torino, Via P. Giuria 9, 10125 Torino, Italy; 2 Institute of Chemistry, University of Tartu, Ravila 14a, 50411 Tartu, Estonia

**Keywords:** *C. argyroa* Biv., *C. cephalanthus* Viv., *C. nutans* subsp*. macrocephalus* (Desf.) Nyman, *C. pycnocephalus* L., *P. casabonae* (L.) Greuter, Asteraceae, primary metabolites, amino compound profiling, DEEMM derivatization

## Abstract

The cardueae are a common species in the Mediterranean area where they grow spontaneously and are traditionally employed as food and for health purposes. In this work, five Cardueae, including two sub-endemic species (four *Carduus* and three *Ptilostemon casabonae* (L.) Greuter samples from different locations) were collected from Sardinia and the Corse islands. All the considered plants are characteristic of the area, in particular the sub-endemic species *C. cephalanthus* and *P. casabonae*. This work aims to obtain, for the first time, the amino compounds profile (primary metabolites) of these little-studied species to detect for any similarities and differences among the different samples using statistical analyses. A recently developed method was employed, where diethyl ethoxymethylenemalonate (DEEMM) derivatives are detected in a neutral loss scan mode using high performance liquid chromatography in tandem with a mass spectrometry technique. In total, 42 amino compounds were detected, of which 33 were fully identified and semi-quantified. Overall, the results show that DEEMM-derivatized amino compounds are qualitatively similar among the considered samples. Nonetheless, a discrimination at the genus level is possible. This work adds more information regarding the phytochemical composition regarding the primary metabolites of the considered samples, their discriminations and the search for compounds with potential health benefits.

## 1. Introduction

Species belonging to the Cardueae tribe (Asteraceae family, Carduoidae subfamily) are annual or biennial herbs characterized by the presence of thorns on the leaves, stems and involucral bracts. Their flowers are purple, rarely pink and the fruit is achene with pappus. It is common to find these species in the Mediterranean landscape, especially in dry and open habitats, abandoned agricultural fields, ruins and roadsides, although they can grow in different continents and ecosystems, such as steppes [1,2,3,4]. These plants are commonly known as thistles and are traditionally employed, in different cultures, for both food and medicinal purposes [5,6,7]. Their taste and their healthy properties for different ailments are due to the presence of different classes of specialized metabolites, known for their beneficial activities (e.g., flavonoids, phenolic acids and terpenes) [8,9,10,11]. In this work, five Cardueae species were collected from Sardinia and the Corse islands where their traditional use is widespread. The selected species belong to the Carduinae subtribe and specifically to the *Carduus* (*C. argyroa* Biv., *C. cephalanthus* Viv., *C. nutans* subsp*. macrocephalus* (Desf.) Nyman and *C. pycnocephalus* L.) and *Ptilostemon* (*P. casabonae* (L.) Greuter) genera. They are all wild characteristic edible species that grow spontaneously in Sardinia, where they are traditionally known as Cardu, Baldu, Gardu and are consumed in different recipes, raw or cooked and for the treatment of several ailments, including gastrointestinal and urogenital disorders [6,10,12,13].Additionally, *C. cephalanthus* and *P. casabonae* are sub-endemic species. *C. cephalanthus* grows selectively in Sardinia, Corse, Sicily, the Tuscan archipelago and Algeria, while *P. casabonae* is present in Sardinia, the Corse and in the Hyères islands (France) [1,14,15,16]. Studies on the phytochemical composition of the extracts obtained from these species are focused on specialized metabolites, especially polyphenols (i.e., flavonoids and phenolic acids) [10,11,17,18]. Few data on amino acids detected in closely related species are documented, e.g., *Carduus acanthoides* L. and *Cynara cardunculus* L. [19,20,21]. Our previous studies [10,11] on the same *Carduus* and *Ptilostemon* species focused on the evaluation of the polyphenolic and biomolecular profiles, which were combined to obtain a useful fingerprinting for taxonomic studies. In this contribution, we aimed at analyzing the extracts obtained from the five Cardueae species to elucidate their amino compounds profile and to improve the knowledge of these little-studied plants. For this purpose, a recently developed method for amino compounds detection, characterized by a prior derivatization step and a consequent analysis through high performance liquid chromatography coupled in tandem to a mass spectrometric detector (HPLC-MS/MS) was applied [22]. In this approach, which was called derivatization-targeted analysis, diethyl ethoxymethylenemalonate (DEEMM) was employed as the derivatization reagent, the reaction was quenched with hydroxylamine (NH_2_OH), and the analysis was carried out through HPLC-MS/MS in a neutral loss scan mode (NLS). The DEEMM derivatives are characterized by a characteristic fragmentation pattern of a precursor ion, which corresponds to the loss of a neutral ethanol molecule [M+H-46]^+^, which can be detected in the NLS mode. This new method was employed to highlight and evaluate, for the first time, the presence of known and unknown amino compounds in the plant’s extracts, which are relevant from a food and therapeutic point of view. This work aims to test its feasibility in a complex study with several plant species to obtain a comprehensive chemical characterization of these plants, including their primary metabolites (e.g., amino acids and biogenic amines), supporting their traditional employment and promoting their consumption. Furthermore, the characterization of the investigated extracts for their content of amino compounds is exploited on the basis of any potential similarities and differences in the profile of the amino compounds among the different species and the geographical localities using a principal component analysis (PCA), and a partial least-squares discriminant analysis (PLS-DA) as an additional fingerprint based upon the primary metabolites.

## 2. Results and Discussion

### 2.1. Phytochemical Analyses

#### 2.1.1. Optimization of the Extraction Procedure

The most common extraction method for amino compounds from solid samples is ultrasound-assisted solid-liquid extraction using different solvents, as reported in the literature, such as 0.1% aqueous formic acid [23], methanol:0.1 M aqueous HCl (88:12) [24], 0.4 M perchloric acid [25], 0.1 M HCl [26] and 0.1 M HCl with 0.2% 3,3′-thiodipropionic acid [27,28].

Some important parameters for an effective extraction process, apart from the type of solvent used, include the volume of solvent, the amount of solid sample, the extraction time and the temperature [29]. *Carduus nutans* subsp*. macrocephalus*, employed as a case study by [22], was here selected to perform all the extraction optimization experiments by applying a Design of Experiment (DoE) using the peak areas’ sum of the amino compounds as the output variable (response). As reported in [22], extraction with 0.1 M HCl in 30% methanol, ethanol or acetonitrile did not show any significant differences in the amino compound profile. The employment of other solvents, e.g., organic solvents without HCl, water or 0.1% formic acid, did not improve the selective extraction of these compounds (data not shown). Therefore, 0.1 M HCl in 30% methanol was selected as the extraction solvent for all the further analyses. In order to find the optimum parameters for the extraction of the amino compounds, a screening experiment was designed to identify any statistically significant parameters among the volume of solvent (set to 3–10 mL), the amount of solid sample (set to 50–200 mg) and the extraction time (set to 10–40 min). Room temperature was selected for all the experiments since higher temperatures can promote thermolabile compounds degradation [29]. The preliminary results showed that the only parameter that was not statistically significant in the sum of the peak areas of all the detected compounds was the extraction time (Figure S1), which was set at 10 min in the following experiments for optimization.

A central composite design (CCD) was employed for the optimization of the extraction procedure with regards to the volume of the solvent and the amount of plant matrix (Table S1) [30]. The response surface (Figure S2) shows that a higher sum of peak areas is obtained with an increasing amount of plant material and a decreasing amount of solvent volume. Therefore, the plant amount chosen was 200 mg with a solvent volume of 3 mL. The repeatability (*n* = 3) of the extraction was evaluated by the relative standard deviation (RSD) values. For most of the peak areas, RSD remained below 15%.

#### 2.1.2. DEEMM Derivatization-Targeted Characterization by HPLC-MS/MS

**Figure 1:**
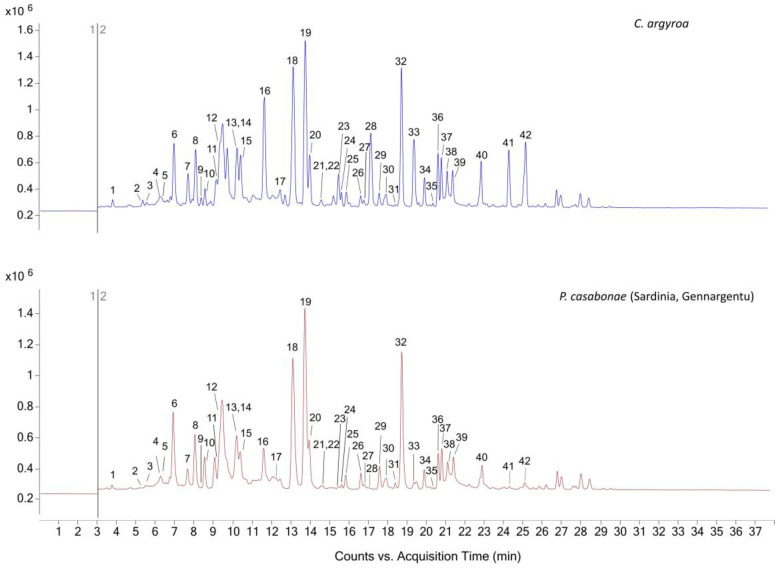
Comparison of NLS mode profiles of DEEMM derivatives in *C. argyroa* and *P. casabonae* from Sardinia (Gennargentu). Compound peak numbers refer to Table 1.

The optimized extraction method was employed for the analysis of 42 plant samples from different *Carduus* species and from *P. casabonae* collected from different locations. All the extracts were then submitted to derivatization, the reaction was quenched with NH_2_OH and the DEEMM derivatives were analyzed using HPLC-MS/MS. In order to perform an untargeted analysis of all the compounds that reacted with the derivatization reagent (derivatization-targeted analysis) and to identify or hypothesize known and unknown amino compounds, the MS/MS detector was operated in the NLS mode. Thirty three of the 42 detected compounds were confirmed through the co-injection of the commercial reference standards (Table 1). The profile is qualitatively stable among all the considered samples, a representative comparison between the NLS profiles of *C. argyroa* and *P. casabonae* from Sardinia (Gennargentu) is shown in Figure 1.

Nine additional compounds to those previously detected in *C. nutans* subsp*. macrocephalus* [22] were identified as a result of optimizing the extraction procedure and employing a slower gradient than previously used, thereby providing a better peak separation and allowing for the detection of more compounds. The newly identified compounds, which were reported for the first time in all the considered species, are taurine, histamine, glycine, glutamic acid, methylamine, ethylamine, dopamine, pyrrolidine and isopentylamine. All the derivatives were detected for the first time in all the analyzed extracts from the different species and localities, confirming the similarity of the amino compound profile among Cardueae species.

The analysis of these primary metabolites can be an additional tool for the characterization of closely related species from the same tribe or of samples from different geographical origins. The literature reports the discrimination of plant samples from different species, cultivars or geographical origins based on the analysis of their amino compound profiles [31,38,39,40]. Moreover, amino acids and other amino compounds are important for their nutritional and medicinal properties. All the investigated thistles are traditionally used for food purposes, the content of amino compounds, especially essential amino acids, is therefore very important since their intake is critical for human metabolism. Among the notable non-amino acid compounds, taurine is mostly found in high quantities in animal products while plants are a poor source [32,41,42], which makes this studied species an interesting source of taurine for vegans, for example. Moreover, some amino compounds have secondary metabolic roles, for instance, glutamine is involved in the pH homeostasis in the liver, while serine is employed in the treatment of some neurological diseases and several proteins and non-protein amino acids are neurotransmitters [43]. This adds value to these species by supporting their traditional therapeutic uses based on their phytochemical composition.

### 2.2. Statistical Analysis

In light of the above considerations and based on the previous results obtained on the discrimination of these species considering their phenolic profile [10,11] statistical data treatments were performed to emphasize any potential similarities and differences of the derivatized amino compound profiles among the different *Carduus* species and *Ptilostemon casabonae* from different geographical origins. The unsupervised principal component analysis (PCA), performed to evaluate the grouping of the investigated samples, was followed by a targeted supervised analysis (partial least-squares discriminant analysis, PLS-DA) and the presence of any statistically significant differences among the samples were evaluated by the analysis of variance (ANOVA). For this purpose, the peak areas of 41 detected compounds in the NLS mode (out of 42) were employed as variables. β-Aminobutyric acid (BABA) was not considered in the statistical analysis due to coelution issues.

**Figure 2:**
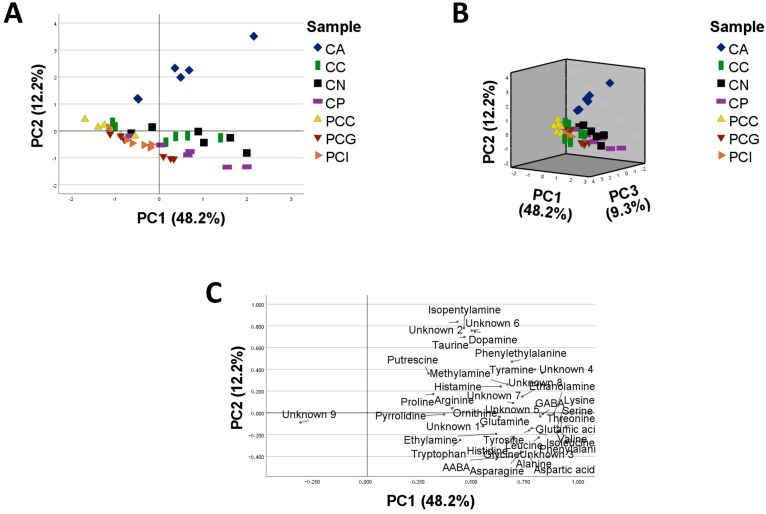
Principal Component Analysis (PCA) of 42 Cardueae samples (six individuals for each species/locality) based on peak areas in NL scan mode of 41 informative compounds selected as variables: (**A**) score plot of the first principal component against the second component, (**B**) 3D score plot of the first, second and third principal components (**C**) loading plot of the variables. CA: *C. argyroa*; CC: *C. cephalanthus*; CN: *C. nutans* subsp*. macrocephalus*; CP: *C. pycnocephalus*; PCC: *P. casabonae* from Corse; PCG: *P. casabonae* from Sardinia (Gennargentu); PCI: *P. casabonae* from Sardinia (Iglesias).

Concerning the PCA, the first, second and third component explain 48.2%, 12.2% and 9.3% of the variability, respectively. The species belonging to the *Carduus* genus are, with some exceptions, more positively correlated with the first component when compared to the samples belonging to the *Ptilostemon* genus (Figure 2A,B). Considering the second component (Figure 2A), the species *C. argyroa* is more separated from the other samples, while the third component does not provide any additional information for the discrimination of the samples according to their amino compound profiles (Figure 2B).

The loading plot (Figure 2C) shows the distribution of the variables. Most of the compounds show higher weights in PC1 with values below 1, the only exception is the unknown 9 which has a negative weight in PC1. This distribution may explain the separation of the *Carduus* species from *P. casabonae*, in particular, unknown 9 could be a discrimination marker for the *P. casabonae* species. On the other hand, unknown 2, isopentylamine, unknown 6, dopamine and taurine are positively correlated with PC2 and can be responsible for the discrimination of *C. argyroa* individuals.

**Figure 3:**
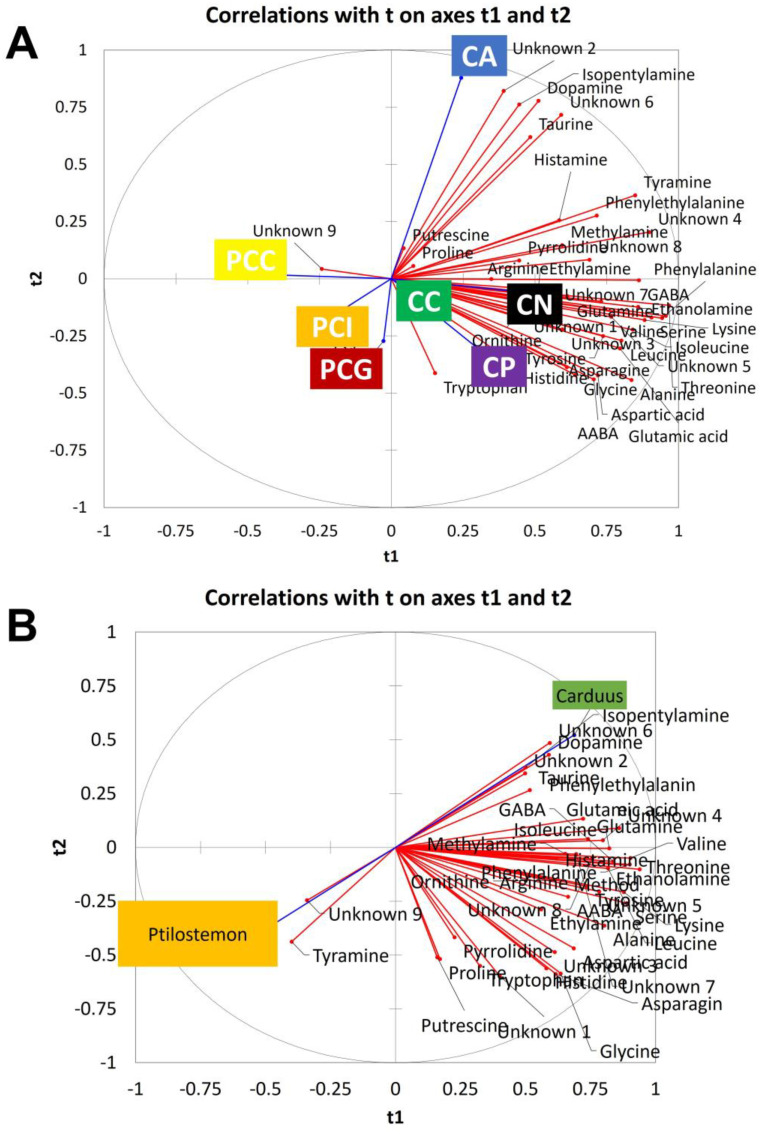
Partial least-squares discriminant analysis (PLS-DA) 42 Cardueae samples (six individuals for each species/locality) based on peak areas in NL scan mode of 41 informative compounds selected as variables: (**A**) score plot of samples grouped by the species/locality of belonging (**B**) score plot of samples grouped by the genus of belonging. CA: *C. argyroa*; CC: *C. cephalanthus*; CN: *C. nutans* subsp*. macrocephalus*; CP: *C. pycnocephalus*; PCC: *P. casabonae* from Corse; PCG: *P. casabonae* from Sardinia (Gennargentu); PCI: *P. casabonae* from Sardinia (Iglesias).

PLS-DA was applied by grouping the samples based on the species for the *Carduus* genus and on the geographical origin for the *P. casabonae* samples. The results of this supervised statistical analysis confirmed a slight separation of the *P. casabonae* samples from the *Carduus* samples based on the t1 component and a discrimination of *C. argyroa* from the other species on the t2 component (Figure 3A).

The distribution of the variables is confirmed as well since it supports the loading plot obtained with the PCA. Figure S3A reports the compounds which give a higher contribution to the variance explanation (variables importance in the projection (VIP) values ± standard deviation (SD) higher than 1) [44], which were considered in the discrimination of each class against the others, with some of these compounds being selected for a box plot representation (Figure S3B). The variability of the considered compounds is, in some cases, substantial. For example, tyramine is more abundant in *Carduus* sp. samples when compared to the extracts obtained from *P. casabonae* from Sardinia and Corse. This difference was confirmed to be statistically significant using the ANOVA analysis (*p* < 0.05). Moreover, some differences can be detected among the different samples, for example isoleucine is more abundant in the *C. pycnocephalus* samples when compared to the other *Carduus* sp. (*p* < 0.05). The alanine content is lower in the *P. casabonae* from Corse (*p* < 0.05) when compared to the samples of the same species collected in Sardinia, while valine was more abundant in *P. casabonae* from Gennargentu when compared to the other two localities (*p* < 0.05). These findings show that few differences in the DEEMM-targeted amino compound profile can be employed for discrimination purposes among the different species of the same genus and in the samples of the same species from different geographical origins.

In order to highlight the differences among the two considered genera, a new PLS-DA was performed where all the *Carduus* species were grouped together as well as the *P. casabonae* from the different geographical areas. As reported in Figure 3B, the separation of the samples based on the t1 component and on the basis of their genus of belonging is confirmed. The variability of some of the compounds, with variable importance in the projection (VIP) values ± standard deviation (SD) higher than 1 (Figure S4A), was illustrated using a box plot. As shown in Figure S4B, unknown 4, unknown 6 and dopamine are more abundant in the *Carduus* species when compared to the *Ptilostemon* samples. This difference is significant from a statistical point of view (Student’s t-test, *p* < 0.05), and supports the use of the DEEMM-targeted amino compound profiling for the discrimination of *Carduus* species from *P. casabonae*.

**Figure 4:**
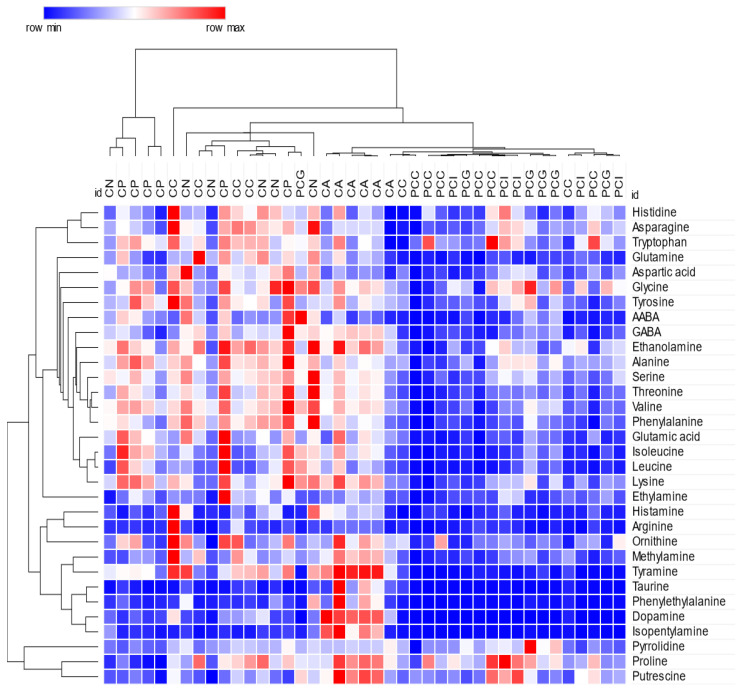
Hierarchical clustering based on Pearson distances and heat-map visualization (42 samples × 32 compounds).

To better estimate the concentrations of these compounds in the investigated samples, a semi-quantitative analysis was performed for all the identified compounds (Table S2). The concentration values were calculated based on a single-point external calibration method. As already discussed, the amino compound content in the *Cardueae* extracts is characterized by a high variability and these results are supported by the literature data [45,46,47]. In general, all the samples proved to be rich in proline, glutamine, glutamic acid and asparagine. On the other hand, the less abundant compounds were the biogenic amines methylamine, histamine, ethylamine, phenylethylamine and isopentylamine. The quantitative analysis was not performed for BABA due to its co-elution with unknown 5;therefore, not allowing for the accurate integration of its peak. However, the presence of BABA was more evident in the *P. casabonae* samples. The visualization of the obtained quantitative results in the form of a heatmap graph confirms a clustering of the samples based on their genus of belonging, with few exceptions (Figure 4). Moreover, it is evident that the extracts obtained from the *Carduus* species show a richer profile and could therefore be considered a better source of amino compounds. A comparison with the literature data shows that the content of some characteristic essential amino acids in the investigated species is comparable to those observed, for example, in *Aloe sp.*, pumpkin andbanana for arginine and strawberry for both valine and phenylalanine [46]. The concentration of other amino compounds that were found in high amounts in the *Cardueae* extracts, including glutamine, glutamic acid and asparagine, is in line with the literature data on the amino acid content in different types of teas [48], while the proline content is comparable to those observed, for example, in *Panax ginseng* C.A.Mey., *Passiflora incarnata* L. and tobacco leaves [31,45]. Concerning the biogenic amines, the histamine content is similar to that found in tomato, pumpkin and pineapple juice [47], while the taurine concentration is similar to the one found in onion bulbs [41].

## 3. Materials and Methods

### 3.1. General Experimental Procedures

HPLC-grade acetonitrile and methanol were from Merck. Hydrochloric acid (HCl), glycine and hydroxylamine (>97.5%) were purchased from Reakhim. DEEMM and L-ornithine monohydrochloride (≥99%) was obtained from Fluka. Boric acid (≥99%) was purchased from Hopkin & Williams. Amino acid commercial standards (L-alanine, L-arginine hydrochloride, L-asparagine, L-aspartic acid, L-glutamine, L-glutamic acid, L-glycine, L-histidine, L-isoleucine, L-leucine, L-lysine, L-methionine, L-phenylalanine, L-proline, L-serine, L-threonine, L-tryptophan, L-tyrosine and L-valine, ≥99.5%), γ-aminobutyric acid (≥99%), formic acid (≥95%), putrescine (≥99.5%), phenyethylamine (≥99.5%), tyramine hydrochloride (≥98%), ethylamine hydrochloride (98%), methylamine solution (40%), pyrrolidine (99%), ethanolamine (≥98%) and taurine (≥99%) were purchased from Sigma; dopamine hydrochloride (99%), histamine dihydrochloride (≥98%) were purchased from Alfa Aesar; L(+)-α-aminobutyric acid (98%) were purchased from Acros Organics; β-aminobutyric acid (98%) were purchased from BLD Pharmatec. Ultrapure water was supplied by a Millipore Milli-Q Advantage A10 (Millipore) and 0.75 M borate buffer at pH = 9 was prepared in deionized water.

### 3.2. Plant Material

The aerial parts of the six individuals of each of the five Cardueae species were harvested in spring (May–June), during the flowering stage from different localities in Sardinia and Corse, the details are reported in Table S3 (species, locality and voucher specimen). The collected fresh plants were oven dried at 40 °C until a constant weight and grinded to a homogeneous powder.

### 3.3. Preparation of the Extracts and Pre-Column Derivatization

A Design of Experiment (DoE) was carried out to optimize the amount of plant material, solvent volume and extraction time; *C. nutans* subsp*. macrocephalus* was selected as a representative species for the experimental design tests. The optimized extraction procedure consists of the addition of 3 mL of 0.1 M HCl in 30% methanol to 200 mg of powdered plant material followed by 10 min in an ultrasonic bath (Bandelin Sonorex) at room temperature and a centrifugation step of 10 min at maximum speed (MTS MPW 340 centrifuge). The supernatant was brought to a volume of 3 mL and filtered with a 25 mm diameter, 0.20 μm pore diameter hydrophilic regenerated cellulose syringe filter Chromafil^®®^Xtra. An aliquote of the obtained extract was then derivatized following the procedure reported by [22].

### 3.4. HPLC-MS/MS Analysis

The analyses were conducted on an HPLC-MS system with Agilent 1290 Infinity II quaternary pump, column thermostat, an autosampler and an Agilent 6460 Triple Quadrupole (QqQ) mass spectrometer (MS) with Agilent Jet Stream Technology (heated electrospray) ionization source (ESI), as reported by [22] with slight modifications. A Zorbax Eclipse Plus C18 (3.0 × 100 mm, 1.8 μm) column was employed and 0.1% aqueous formic (A) and acetonitrile (B) were used as mobile phases. The gradient program for the analyses was: 0–2 min, 10% B; 2–13 min, 10–25% B; 13–32 min, 25–75% B; 32–35 min, 75–100% B, 35–37 min, 100% B and 37–38 min, 100–10% B; the total cycle time was 43 min; flow rate: 0.5 mL/min. The ESI and MS parameters: drying gas temperature 320 °C, drying gas flow 9 L/min, nebulizer gas pressure 45 psi, sheath gas flow 12 L/min, sheath gas temperature 400 °C, capillary voltage 3000 V and nozzle voltage 0 V. Neutral loss scan mode was performed with the neutral loss of 46 in the *m/z* range from 50 to 600, collision energy 8 V and fragmentor 90 V. The data was processed using the Agilent MassHunter Qualitative Analysis Navigator B.08.00 software. The quantification of the detected compounds was performed in the NLS mode employing the single-point external calibration method.

### 3.5. Statistical Analysis

All the statistical analyses were performed using the peak areas obtained in the NLS mode as variables.

The design of experiment (DoE) was carried out on R software Chemometric Agile Tool (CAT) [49].

The principal Component Analysis (PCA), the student’s t-test and the ANOVA analysis (*p* < 0.05) were carried out using SPSS 28.0 (IBM Corporation). The partial least-squares discriminant analysis (PLS-DA) was performed on XLSTAT statistical. Peak areas were employed as input variables.

The heatmap was created by using Morpheus software [50].

## 4. Conclusions

In this work,, reported for the first time, the derivatization-targeted analysis of the amino compounds in five selected plant species belonging to the Cardueae tribe and including two sub-endemic species (*C. cephalanthus* and *P. casabonae*) was performed. The neutral loss scan mode in the HPLC-MS/MS after the DEEMM derivatization and NH_2_OH quenching enabled the untargeted detection of 42 distinctive peaks, including known and unknown amino compounds of which 33 were fully identified. The amino compound profile was compared across the 42 plant samples, with four species belonging to the *Carduus* genus and the *Ptilostemon casabonae* samples from the different locations in the Mediterranean area. Overall, all the considered samples are characterized by the presence of the same amino compounds, no qualitative differences are detectable. The PCA, PLS-DA and ANOVA analyses showed some statistically significant differences in the amount of some of the compounds, especially among the two considered genera. Few differences were detected among the four *Carduus* species or among the three *P. casabonae* samples from the different localities. These findings add more information to the previous studies on the phenolic profiles of the same species considered in this work. The qualitative similarity in the amino compound profiles makes these species good candidates for food supplements and functional food, with a special interest in the *Carduus* species. This approach enables the detection of interesting primary metabolites with nutritional values and biological activities, which supports the traditional employment of these plants for alimentary and medicinal applications and to promote their use.

## Figures and Tables

**Table 1:** Identified amino compounds in *Carduus* species and *Ptilostemon casabonae*. Each peak is characterized by retention time, ESI^+^ protonated molecule (*m/z*), DEEM derivative and corresponding amino compound nominal mass (g/mol) and compound names. All the identified compounds were confirmed by the injection of reference standards.

N°	Retention Time (min)	ESI^+^ Protonated Compound (m/z)	Derivative Nominal Mass (g/mol)	Nominal Mass (g/mol)	Compound Name	Reference
1	3.78	326	325	155	Histidine	[31]
2	5.35	296	295	125	Taurine	[32]
3	5.54	282	281	111	Histamine	[33]
4	6.24	345	344	174	Arginine	[31]
5	6.32	365	364/342 ^1^	194/172 ^1^	Unknown 1	
6	6.97	303	302	132	Asparagine	[31]
7	7.70	317	316	146	Glutamine	[31]
8	8.10	276	275	105	Serine	[31]
9	8.40	301	300/278 ^1^	130/108 ^1^	Unknown 2	
10	8.59	259	258/236 ^1^	88/66 ^1^	Unknown 3	
11	9.20	304	303	133	Aspartic acid	[31]
12	9.37	232	231	61	Ethanolamine	
13	10.24	246	245	75	Glycine	[31]
14	10.28	318	317	147	Glutamic acid	[31]
15	10.45	290	289	119	Threonine	[31]
16	11.68	260	259/237 ^1^	89/67^1^	Unknown 4	
17	12.51	202	201	31	Methylamine	[34]
18	13.20	274	273	103	γ-aminobutyric acid (GABA)	[31]
19	13.83	260	259	89	Alanine	[31]
20	14.07	286	285	115	Proline	[31]
21	14.66	274	273/251 ^1^	103/81^1^	Unknown 5	
22	14.70	274	273	103	β-aminobutyric acid (BABA)	[35]
23	15.57	288	287/265 ^1^	117/95 ^1^	Unknown 6	
24	15.73	440	439	269	Unknown 7	
25	15.97	352	351	181	Tyrosine	[31]
26	16.73	274	273	103	α-aminobutyric acid (AABA)	[35]
27	16.89	216	215	45	Ethylamine	[34]
28	17.26	324	323	153	Dopamine	[36]
29	17.70	242	241	71	Pyrrolidine	[37]
30	18.06	288	287/265 ^1^	117/95 ^1^	Unknown 8	
31	18.51	301	300/278 ^1^	130/108 ^1^	Unknown 9	
32	18.86	288	287	117	Valine	[31]
33	19.47	308	307	137	Tyramine	[31]
34	20.05	375	374	204	Tryptophan	[31]
35	20.72	427^2^	472	132	Ornithine	[31]
36	20.76	336	335	165	Phenylalanine	[31]
37	20.93	302	301	131	Isoleucine	[31]
38	21.24	302	301	131	Leucine	[31]
39	21.54	441 ^2^/509 ^3^	440	270	Lysine	[31]
40	23.02	383	382	212	Putrescine	[31]
41	24.47	292	291	121	Phenylethylamine	[31]
42	25.35	258	257	87	Isopentylamine	[31]

^1^ nominal mass without or with sodium adduct, ^2^ Loss of 46 from the disubstituted derivative, ^3^ Sodium adduct [M+Na]^+^.

## Data Availability

Not applicable.

## Supplementary Materials

The following supporting information can be downloaded at: https://www.mdpi.com/article/10.3390/plants12020319/s1, Table S1: Coded and natural variables on the considered parameters and design matrices for the optimization of the extraction; Table S2: Average values of concentration (mg/kg) in the plant material from six individuals of each of the four *Carduus* species and three different collection areas of *Ptilostemon casabonae*. The numbers in parenthesis represent the lowest and highest value of concentration. (CA: *C. argyroa*; CC: *C. cephalanthus*; CN: *C. nutans* subsp. *macrocephalus*; CP: *C. pycnocephalus*; PCC: *P. casabonae* from Corse; PCG: *P. casabonae* from Sardinia-Gennargentu; PCI: *P. casabonae* from Sardinia-Iglesias); Table S3: Sites, and voucher numbers, five Cardueae species; Figure S1: Pareto Chart of the preliminary DoE showing the significance of each variable (extraction time, solvent volume and the amount of plant matrix) of the response surface model on the sum of the peak areas; Figure S2: Response surface based on the sum of the peak areas of the DEEMM-derivatives found in the extracts obtained through the Central Composite Design (CCD).; Figure S3: (A) Variable importance in the projection (VIP) for the discrimination of the *Carduus* species and the *Ptilostemon* samples from the different geographical areas. (B) Box plot of some compound’s selected considering their VIP value to compare their variability in the *Carduus* species and the *Ptilostemon* samples from the different geographical areas. CA: *C. argyroa*; CC: *C. cephalanthus*; CN: *C. nutans* subsp. *macrocephalus*; CP: *C. pycnocephalus*; PCC: *P. casabonae* from Corse; PCG: *P. casabonae* from Sardinia (Gennargentu); PCI: *P. casabonae* from Sardinia (Iglesias).; Figure S4: (A) Variable importance in the projection (VIP) for the discrimination of *Carduus* and *Ptilostemon* samples at the genus level. (B) Box plot of some compounds selected considering their VIP value to compare their variability in the *Carduus* and *Ptilostemon* samples at the genus level.
