# Supplementary material for: Free Amino Acids and Biogenic Amines Profiling and Variation in Wild and Sub-Endemic Cardueae Species from Sardinia and Corse
Source: Plants (Basel). 2023 Jan 10;12(2):319. doi: 10.3390/plants12020319 (PMC9864185; doi:10.3390/plants12020319)
Supplement: Supplementary file 1 [file plants-12-00319-s001.zip › plants-2147123-supplementary.pdf]

Supplementary materials

Article title: Free Amino Acids and Biogenic Amines Profiling and Variation in Wild and Sub-Endemic Cardueae Species from Sardinia and Corse

Journal name: Plants

Author names: Arianna Marengo[a§*], Larissa Silva Maciel[b§], Cecilia Cagliero[a], Patrizia Rubiolo[a], Koit Herodes[b]

Affiliations and e-mail address of the corresponding author:

[a] Dipartimento di Scienza e Tecnologia del Farmaco, Università di Torino, Via P. Giuria 9, 10125 Torino, Italy

[b] Institute of Chemistry, University of Tartu, Ravila 14a, Tartu, 50411, Estonia

[§] These authors gave an equivalent contribution to this work

*Corresponding author: Arianna Marengo: Dipartimento di Scienza e Tecnologia del Farmaco, Universitàdi Torino, Via P. Giuria 9, 10125, Torino, Italy. email:arianna.marengo@unito.it

Larissa Silva Maciel: Institute of Chemistry, University of Tartu, Ravila 14a, Tartu, 50411, Estonia. e-mail: larissasilvamaciel@gmail.com

Table S1. Coded and natural variables on the considered parameters and design matrices for the optimization of the extraction.

	Independent variables	Levels				
		-α	-1	0	+1	+α
Optimization DoE	Solvent volume (mL)	2.6	3	4	5	5.4
	Amount of plant matrix (mg)	128	140	170	200	212

Table S2. Average values of concentration (mg/kg) in the plant material from six individuals of each of the four *Carduus* species and three different collection areas of *Ptilostemon casabonae*. The numbers in parenthesis represent the lowest and highest value of concentration. (CA: *C. argyroa*; CC: *C. cephalanthus*; CN: *C. nutans* subsp. *macrocephalus*; CP: *C. pycnocephalus*; PCC: *P. casabonae* from Corse; PCG: *P. casabonae* from Sardinia-Gennargentu; PCI: *P. casabonae* from Sardinia-Iglesias)

Compound	CA	CC	CN	CP	PCC	PCG	PCI
Histidine	22.71 (5.65-45.6)	29.60 (6.33-63.8)	32.29 (10.2-47.3)	27.13 (9.12-46.6)	22.08 (6.73-38.2)	19.47 (9.83-28.0)	28.09 (11.6-49.8)
Taurine	26.69 (7.93-65.2)	5.64 (1.96-10.2)	6.53 (1.50-11.6)	3.04 (1.29-7.72)	1.01 (0.48-1.20)	1.04 (0.81-1.40)	1.08 (0.68-1.69)
Histamine	0.77 (0.23-1.35)	0.48 (0.09-1.84)	0.89 (0.24-1.50)	0.29 (0.16-0.43)	0.27 (0.10-0.45)	0.17 (0.10-0.22)	0.22 (0.15-0.42)
Arginine	99.58 (33.0-132.5)	149.76 (26.1-561)	65.45 (19.6-92.6)	70.99 (49.0-98.1)	46.03 (11.2-90.8)	38.83 (21.6-59.5)	44.56 (15.7-75.9)
Asparagine	278.24 (60.5-360)	434.08 (91.1-757)	465.56 (156-758)	364.16 (162-523)	229.98 (59.3-481)	279.83 (145-432)	370.82 (183-499)
Glutamine	459.95 (177-737)	671.64 (103-1290)	678.44 (412-947)	520.38 (153-918)	125.34 (46.5-274)	221.09 (77.9-583)	190.26 (121-321)
Serine	96.17 (62.1-121)	79.74 (41.9-108)	129.86 (59.1-188)	108.66 (61.5-136)	39.30 (10.8-72.2)	68.13 (38.3-103)	69.73 (26.6-95.5)
Aspartic acid	21.29 (8.73-31.4)	31.24 (12.4-51.8)	49.46 (18.3-82.8)	34.79 (18.0-64.2)	15.57 (3.62-30.7)	23.02 (9.79-39.6)	27.49 (7.32-41.3)
Ethanolamine	11.10 (8.39-15.0)	10.93 (6.18-14.1)	11.10 (5.25-16.9)	11.31 (6.56-14.9)	4.94 (3.39-9.34)	6.60 (4.89-8.08)	8.64 (7.48-10.5)
Glycine	22.27 (17.5-28.9)	18.76 (11.7-33.0)	26.10 (14.6-36.0)	27.60 (12.8-38.2)	11.73 (5.56-26.0)	26.38 (17.1-39.9)	24.25 (20.9-27.8)
Glutamic acid	322.67 (191-539)	268.48 (163-363)	355.77 (217-519)	463.07 (308-647)	155.12 (108-280)	220.87 (143-290)	165.46 (134-211)
Threonine	62.54 (32.8-81.9)	46.27 (18.7-64.7)	80.76 (39.5-125)	77.40 (32.5-119)	12.47 (4.11-30.5)	37.13 (24.3-56.0)	29.66 (17.7-35.7)
Methylamine	2.05 (1.39-2.66)	1.99 (0.73-3.47)	1.09 (0.67-1.38)	1.05 (0.64-1.54)	0.40 (0.15-0.56)	0.82 (0.56-1.18)	0.84 (0.40-1.17)
GABA	117.26 (101-126)	92.49 (58.4-123)	107.19 (83.9-132)	103.26 (59.8-173)	59.05 (51.5-69.5)	86.77 (64.7-117)	75.66 (70.1-81.7)
Alanine	102.53 (85.4-127)	109.52 (77.2-127)	121.59 (81.8-142)	151.60 (101-189)	57.91 (36.2-84.2)	104.11 (84.0-132)	102.73 (68.6-128)

Proline	4466.69 (3087-5760)	3975.85 (2470-5475)	2958.29 (1346-5269)	2118.01 (876-4216)	3610.25 (1593-5787)	3504.63 (2366-4631)	4297.14 (2480-6375)
Tyrosine	25.25 (17.7-38.3)	25.20 (12.3-52.8)	23.21 (8.99-41.5)	35.75 (21.8-54.3)	7.12 (1.30-11.3)	18.49 (9.76-34.1)	19.33 (10.6-31.1)
AABA	89.12 (40.4-174)	99.87 (20.3-180)	176.22 (73.1-316)	220.97 (112-364)	24.34 (11.7-44.5)	157.05 (40.5-406)	56.07 (23.1-102)
Ethylamine	0.58 (0.40-0.72)	0.53 (0.41-0.70)	0.44 (0.23-0.83)	0.75 (0.38-1.49)	0.30 (0.18-0.47)	0.39 (0.29-0.58)	0.55 (0.38-0.76)
Dopamine	113.29 (51.5-152)	29.38 (2.36-77.4)	27.85 (3.35-61.9)	22.73 (12.9-40.1)	0.07 (0.015-0.15)	0.04 (0.013-0.075)	0.040 (0.020-0.065)
Pyrrolidine	5.87 (3.53-7.45)	4.81 (3.38-5.93)	5.37 (3.88-7.04)	4.37 (3.20-7.12)	4.31 (1.28-6.20)	7.38 (3.60-12.6)	4.69 (2.67-6.54)
Valine	95.46 (64.3-116)	81.42 (38.8-105)	119.40 (84.5-157)	123.94 (79.6-173)	21.43 (11.1-32.0)	77.46 (47.3-116)	48.25 (31.9-57.4)
Tyramine	183.67 (102-221)	93.56 (30.8-203)	123.78 (31.7-195)	110.25 (49.8-168)	5.81 (0.81-14.8)	9.10 (1.99-27.0)	5.50 (2.21-9.13)
Tryptophan	43.47 (9.85-81.0)	54.88 (10.6-86.9)	52.77 (28.8-71.6)	67.31 (51.9-81.6)	61.64 (30.3-105)	40.30 (27.8-56.4)	55.72 (37.7-76.2)
Ornithine	5.99 (4.07-9.58)	5.26 (1.68-10.3)	4.32 (1.26-7.87)	6.23 (2.21-9.12)	4.31 (1.87-7.79)	2.94 (1.89-4.29)	3.92 (1.90-6.07)
Phenylalanine	69.76 (39.9-93.1)	76.30 (31.7-98.7)	112.95 (61.2-162)	83.30 (38.6-135)	16.72 (7.83-29.1)	49.74 (22.1-78.4)	31.24 (24.8-38.4)
Isoleucine	45.15 (19.4-69.2)	20.12 (7.87-31.3)	41.00 (20.0-67.0)	72.92 (36.7-99.6)	4.24 (1.52-7.10)	29.74 (9.46-62.7)	10.34 (5.12-17.2)
Leucine	36.87 (18.8-63.9)	18.89 (7.61-34.4)	38.35 (15.0-69.5)	68.17 (25.5-105)	3.58 (1.12-5.69)	27.79 (6.61-73.9)	10.63 (4.18-21.8)
Lysine	45.50 (22.7-63.0)	25.72 (11.9-48.0)	40.37 (18.4-59.7)	55.86 (29.9-72.4)	12.55 (4.21-27.1)	30.20 (10.5-53.4)	26.11 (15.9-32.4)
Putrescine	5.42 (3.15-7.37)	2.79 (2.07-4.20)	2.81 (1.19-3.75)	2.22 (1.32-2.90)	3.49 (2.44-6.13)	3.16 (2.14-4.82)	4.85 (2.89-7.38)
Phenylethylamine	3.74 (1.02-8.42)	0.66 (0.35-1.07)	2.83 (0.52-5.56)	0.92 (0.49-1.76)	0.12 (0.057-0.20)	0.26 (0.10-0.45)	0.15 (0.11-0.18)
Isopentylamine	13.17 (1.34-19.9)	0.84 (0.47-1.32)	3.38 (0.97-5.98)	1.81 (0.90-2.73)	0.33 (0.23-0.54)	0.50 (0.16-1.30)	0.23 (0.17-0.31)

Table S3: Sites, and voucher numbers, five Cardueae species

**Species**	**Site**	**Coordinates**	**Voucher speciment**
Carduus argyroa	Sardinia-Decimomannu	39°17'47.96"N - 8°58'14.95"E	CAG-803
Carduus cephalanthus	Sardinia-Capo Testa	41°14'33.80"N – 9°8'49.25"E	CAG-807
Carduus nutans subsp. *macrocephalus*	Sardinia-Gennargentu	39°57'35.77"N - 9°19'12.46"E	CAG-802
Carduus pycnocephalus	Sardinia-Monte dei Sette Fratelli	39°20'43.60"N – 9°17'43.74"E	CAG-805
Ptilostemon casabonae	Sardinia-Gennargentu	39°53'54.9"N – 9°26'27.9"E	CAG-796

Ptilostemon casabonae	Sardinia-Iglesias	39°21′45.8″N - 8°32′24.0″E	CAG-796
Ptilostemon casabonae	Corse-Bocca di Tana	41°45.791′N; 009°02.300′E	CAG-796

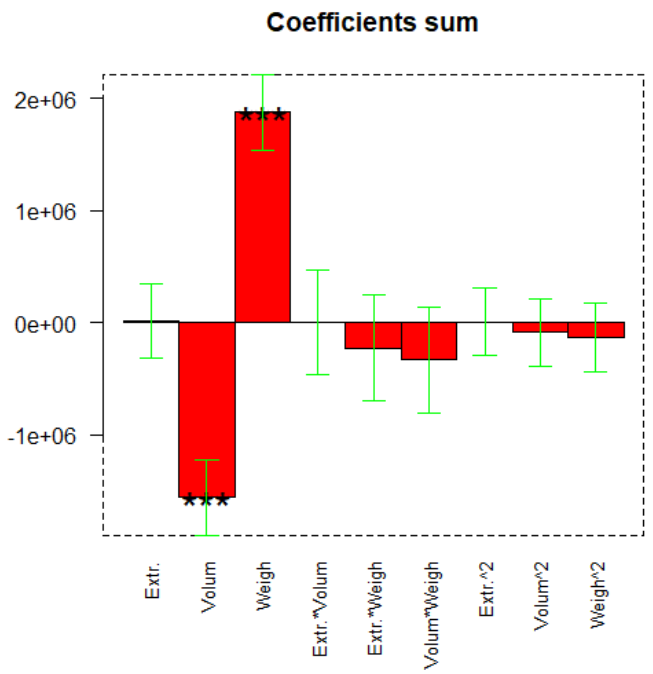

Fig. S1: Pareto Chart of the preliminary DoE showing the significance of each variable (extraction time, solvent volume, and the amount of plant matrix) of the response surface model on the sum of the peak areas

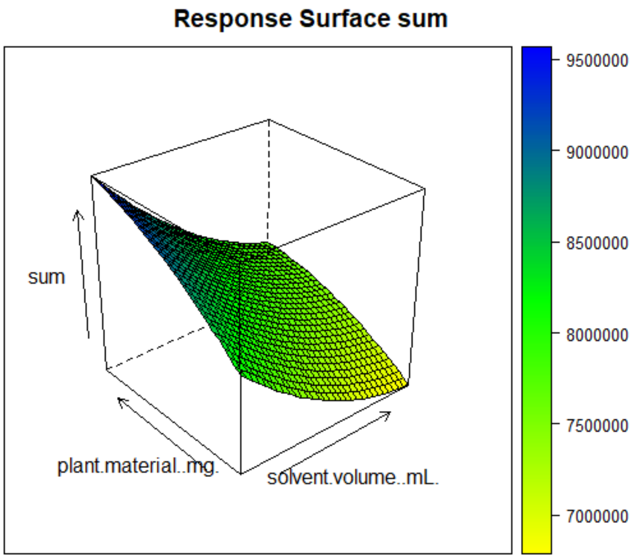

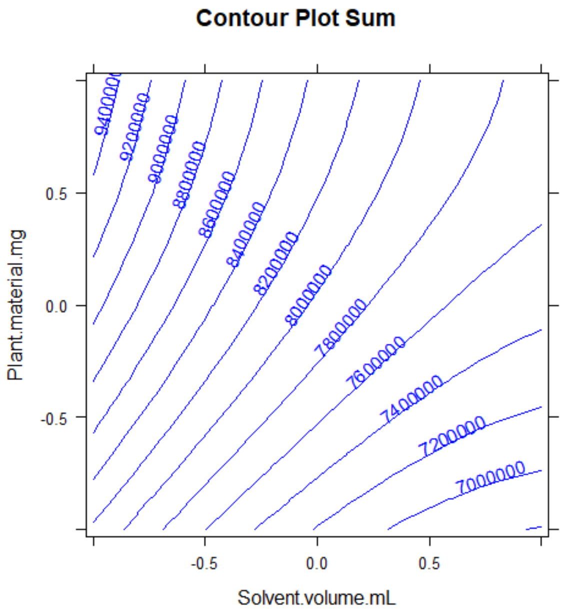

Fig. S2. Response surface based on the sum of the peak areas of the DEEMM-derivatives found in the extracts, obtained through the Central Composite Design (CCD).

A

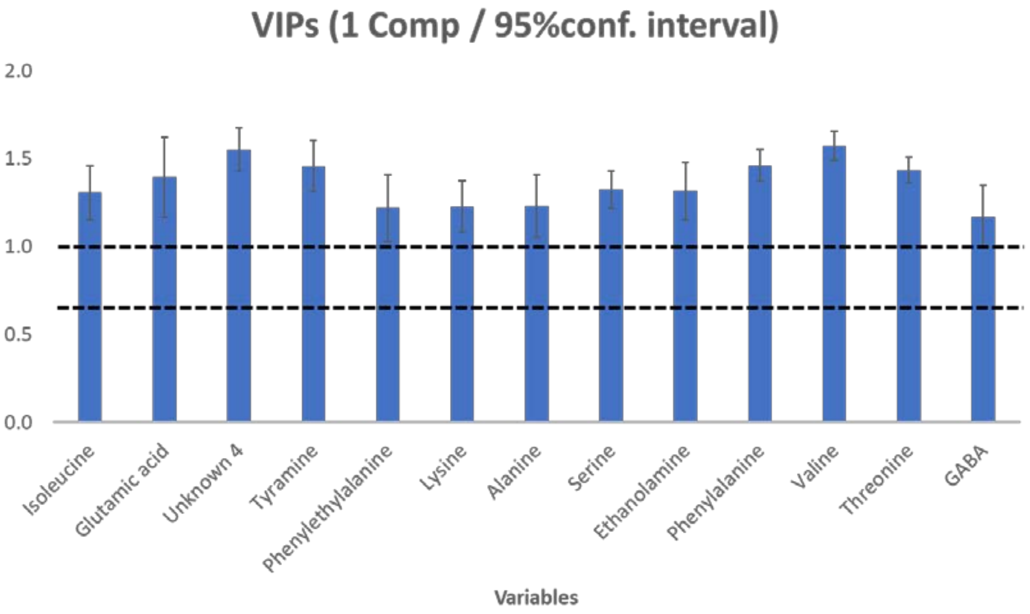

VIPs (1 Comp / 95%conf. interval)
2.0
1.5
1.0
0.5
0.0
Isoleucine
Glutamic acid
Unknown 4
Tyramine
Phenylethylalanine
Lysine
Alanine
Serine
Ethanolamine
Phenylalanine
Valine
Threonine
GABA
Variables

B

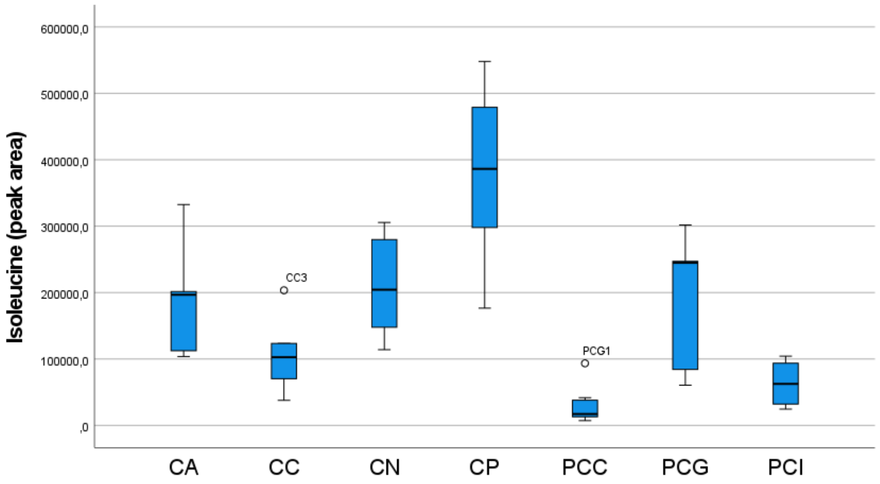

600000,0
500000,0
400000,0
300000,0
200000,0
100000,0
,0
Isoleucine (peak area)
CC3
PCG1
CA
CC
CN
CP
PCC
PCG
PCI

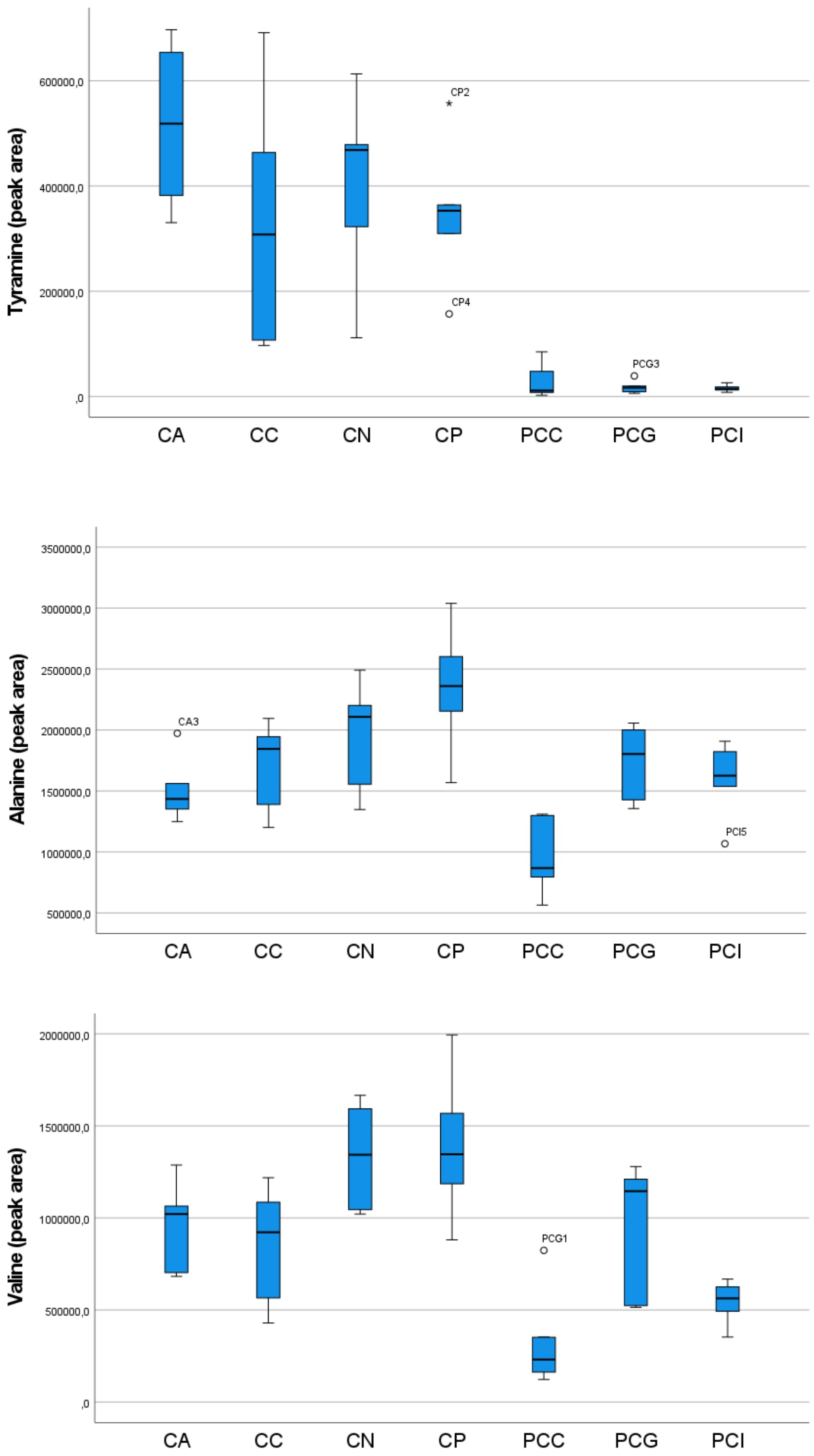

Fig. S3. (A) Variable importance in the projection (VIP) for the discrimination of the *Carduus* species and the *Ptilostemon* samples from the different geographical areas. (B) Box plot of some com-pound's selected

considering their VIP value to compare their variability in the Carduus species and the *Ptilostemon* samples from the different geographical areas. CA: *C. argyroa*; CC: *C. cephalanthus*; CN: *C. nutans* subsp. *macrocephalus*; CP: *C. pycnocephalus*; PCC: *P. casabonae* from Corse; PCG: *P. casabonae* from Sardinia (Gennargentu); PCI: *P. casabonae* from Sardinia (Iglesias).

A

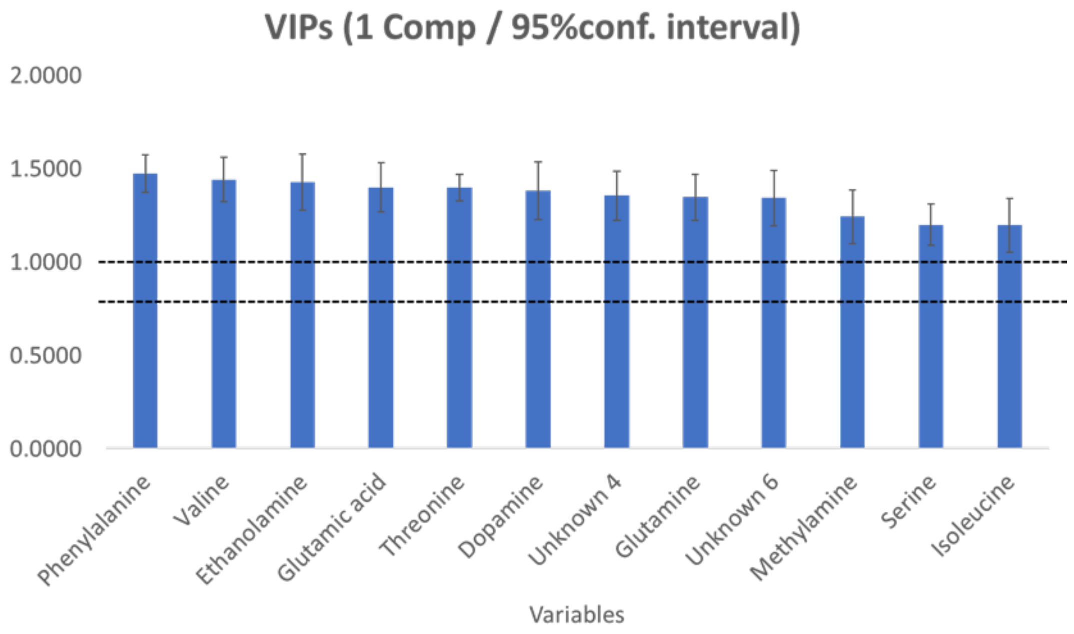

B

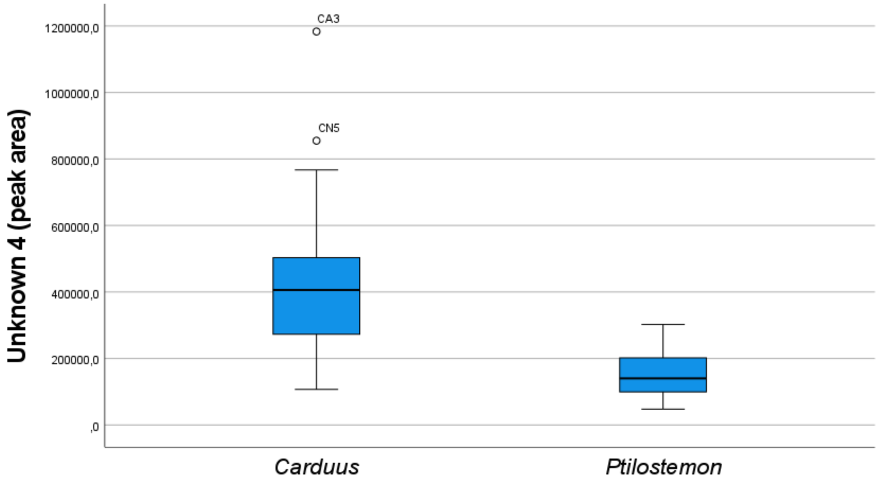

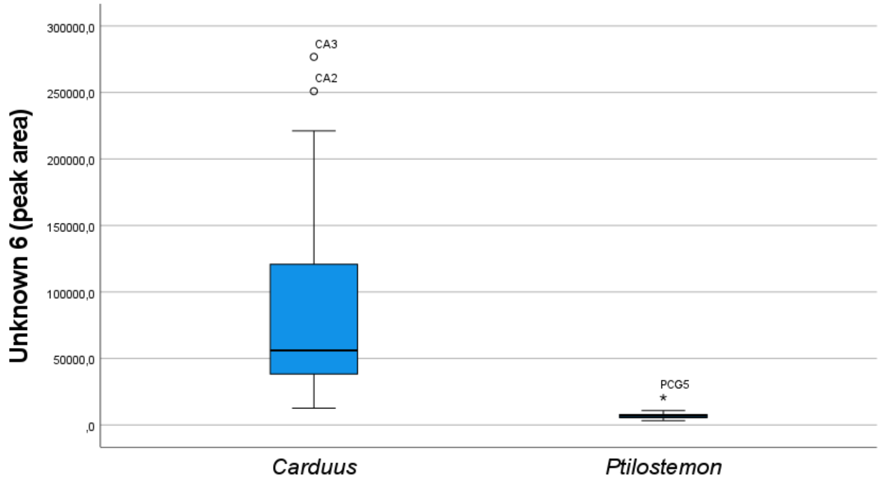

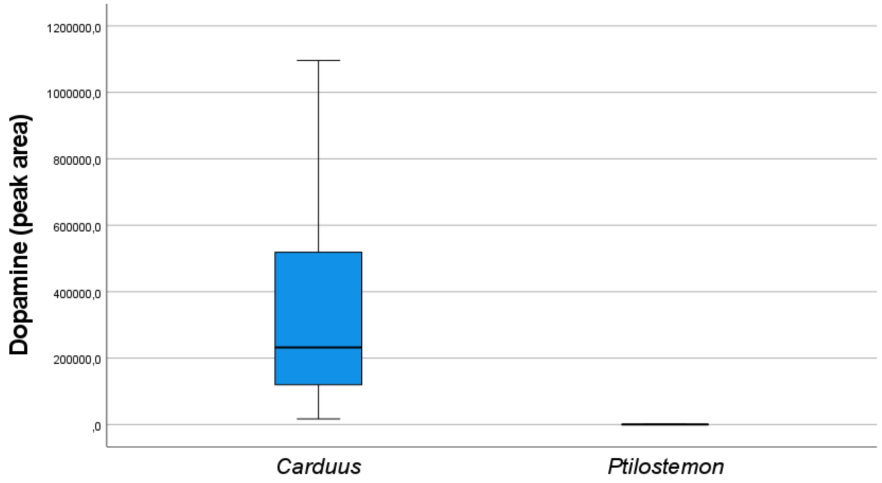

Fig. S4. (A) Variable importance in the projection (VIP) for the discrimination of *Carduus* and *Ptilostemon* samples at the genus level. (B) Box plot of some compounds selected considering their VIP value to compare their variability in the *Carduus* and *Ptilostemon* samples at the genus level.
